# Fetal weight estimation by automated three-dimensional limb volume model in late third trimester compared to two-dimensional model: a cross-sectional prospective observational study

**DOI:** 10.1186/s12884-021-03830-5

**Published:** 2021-05-08

**Authors:** Xining Wu, Zihan Niu, Zhonghui Xu, Yuxin Jiang, Yixiu Zhang, Hua Meng, Yunshu Ouyang

**Affiliations:** Department of ultrasound, Peking Union Medical College Hospital, Chinese Academy of Medical Sciences and Peking Union Medical College, Beijing, 100730 China

**Keywords:** Ultrasound, Estimated fetal weight, Fractional arm volume, Fractional thigh volume, Three-dimensional ultrasound

## Abstract

**Background:**

Accurate estimation of fetal weight is important for prenatal care and for detection of fetal growth abnormalities. Prediction of fetal weight entails the indirect measurement of fetal biometry by ultrasound that is then introduced into formulae to calculate the estimated fetal weight. The aim of our study was to evaluate the accuracy of fetal weight estimation of Chinese fetuses in the third trimester using an automated three-dimensional (3D) fractional limb volume model, and to compare this model with the traditional two-dimensional (2D) model.

**Methods:**

Prospective 2D and 3D ultrasonography were performed among women with singleton pregnancies 7 days before delivery to obtain 2D data, including fetal biparietal diameter, abdominal circumference and femur length, as well as 3D data, including the fractional arm volume (AVol) and fractional thigh volume (TVol). The fetal weight was estimated using the 2D model and the 3D fractional limb volume model respectively. Percentage error was defined as (estimated fetal weight - actual birth weight) divided by actual birth weight and multiplied by 100. Systematic errors (accuracy) were evaluated as the mean percentage error (MPE). Random errors (precision) were calculated as ±1 SD of percentage error. The intraclass correlation coefficient (ICC) was used to analyze the inter-observer reliability of the 3D ultrasound measurements of fractional limb volume.

**Results:**

Ultrasound examination was performed on 56 fetuses at 39.6 ± 1.4 weeks’ gestation. The average birth weight of the newborns was 3393 ± 530 g. The average fetal weight estimated by the 2D model was 3478 ± 467 g, and the MPE was 3.2 ± 8.9. The average fetal weights estimated by AVol and TVol of the 3D model were 3268 ± 467 g and 3250 ± 485 g, respectively, and the MPEs were − 3.3 ± 6.6 and − 3.9 ± 6.1, respectively. For the 3D TVol model, the proportion of fetuses with estimated error ≤ 5% was significantly higher than that of the 2D model (55.4% vs. 33.9%, *p* < 0.05). For fetuses with a birth weight < 3500 g, the accuracy of the AVol and TVol models were better than the 2D model (− 0.8 vs. 7.0 and − 2.8 vs. 7.0, both *p* < 0.05). Moreover, for these fetuses, the proportions of estimated error ≤ 5% of the AVol and TVol models were 58.1 and 64.5%, respectively, significantly higher than that of the 2D model (19.4%) (both *p* < 0.05). The inter-observer reliability of measuring fetal AVol and TVol were high, with the ICCs of 0.921 and 0.963, respectively.

**Conclusion:**

In this cohort, the automated 3D fractional limb volume model improves the accuracy of weight estimation in most third-trimester fetuses. Prediction accuracy of the 3D model for neonatal BW, particularly < 3500 g was higher than that of the traditional 2D model.

## Background

The birth weight (BW) of newborns is intimately related to the maternal and perinatal prognosis. When the BW is too low or too high, morbidity and mortality are significantly higher than those of normal newborns. Fetal growth retardation (FGR) leads to low birth weight (BW < 2500 g) and increases the rate of cesarean section and risk of neurological complications and stillbirth [1–4]. Fetal oversize is associated with prolonged labor and various birth injuries, including shoulder dystocia, brachial plexus injury, and perinatal asphyxia, as well as increased maternal risks, such as obstetric laceration and postpartum hemorrhage [5–7].

In the third trimester, ultrasound measurement of fetal head, abdominal circumference, femoral length and other parameters to estimate fetal weight not only reflects fetal intrauterine nutrition, but also is an important aspect of prenatal care. Hadlock et al. [8] established a fetal weight estimation model using two-dimensional (2D) indexes of fetal head, abdominal circumference, and femur length. With the development of three-dimensional (3D) ultrasound technology, limb volumes have gradually been incorporated into fetal weight estimation models [9–12]. Lee et al. [10] proposed the concept of fractional limb volume, which was added to the new weight prediction models as a fetal soft tissue parameter.

Previous studies have shown that fractional limb volume combined with other 2D biological indicators can improve the precision of fetal weight estimation [11–15]. However, due to the differences in sample size, gestational weeks of ultrasound examination, and ethnicity, the conclusions of different studies vary, and there are relatively few studies on the predictive ability of 3D models for newborns with different birth weights. A commercially available software tool (5D Limb Vol) launched in 2016 can automatically measure the fractional limb volume of the fetus approximately five times faster than manual tracing [16]. Therefore, the main objective of this study was to perform prospective validation regarding accuracy and precision of fetal weight estimation of Chinese fetuses in the third trimester using an automated 3D fractional limb volume model, and to compare this model with the traditional 2D model to determine its clinical application value.

## Methods

### Study population

A cross-sectional prospective observational study was conducted at the Department of Ultrasound in Peking Union Medical College Hospital from January 2018 to July 2019. All patients were identified during their routine clinical care in the prenatal clinic and then invited to participate in a separate research visit.

The inclusion criteria included single live birth, gestational age was calculated according to the last menstrual period and confirmed by ultrasound during the first trimester, and the pregnant women delivered after 34 weeks and delivery date was within 7 days of the last prenatal ultrasound examination. The exclusion criteria were (1) multifetal pregnancy; (2) unclear gestational age; (3) fetal chromosomal abnormality and/or structural malformation; (4) delivery date more than 7 days after the last ultrasound examination.

This study was approved by the Ethics Committee of Peking Union Medical College Hospital, Chinese Academy of Medical Sciences (HS-1420). Before the study was performed, we gave a detailed explanation to the pregnant women and provided written informed consents, and all informed consents were signed by the participants. Neonatal follow-up data included date of delivery, gestational age at delivery, birth weight, and sex.

### Ultrasonographic data acquisition

All 2D biological parameters, including fetal biparietal diameter (BPD), abdominal circumference (AC), femur length (FL), and 3D volume data were acquired using a WS80A ultrasound system (Samsung Medison, Seoul, Korea) by a registered sonographer (XW), who had more than 5 years of experience with 3D ultrasonography. 3D ultrasound scans were performed using a convex volume probe (CV1-8A) to obtain the volume data sets of fetal arm and fetal thigh.

The instrument parameters and processes were set according to previous research regulations [14, 16]. The limbs closest to the anterior uterus wall were selected to display the maximum longitudinal section of the upper limb or lower limb long bone to initiate 3D ultrasound. For each pregnancy, two sonographic volume data sets of the fetal arm and fetal thigh were obtained. The scanning angle was set to 65°- 80° according to different gestational ages with the high quality. When the fetus was large, a wide scan was select to ensure that the sampling frame contained the entire long bone volume of the upper or lower limb.

### Three-dimensional volume data analysis

The best quality imaging were selected for analysis. The optimal imaging refers to no fetal movement artifacts and clear recognition of the soft tissue boundary.

**Fig. 1:**
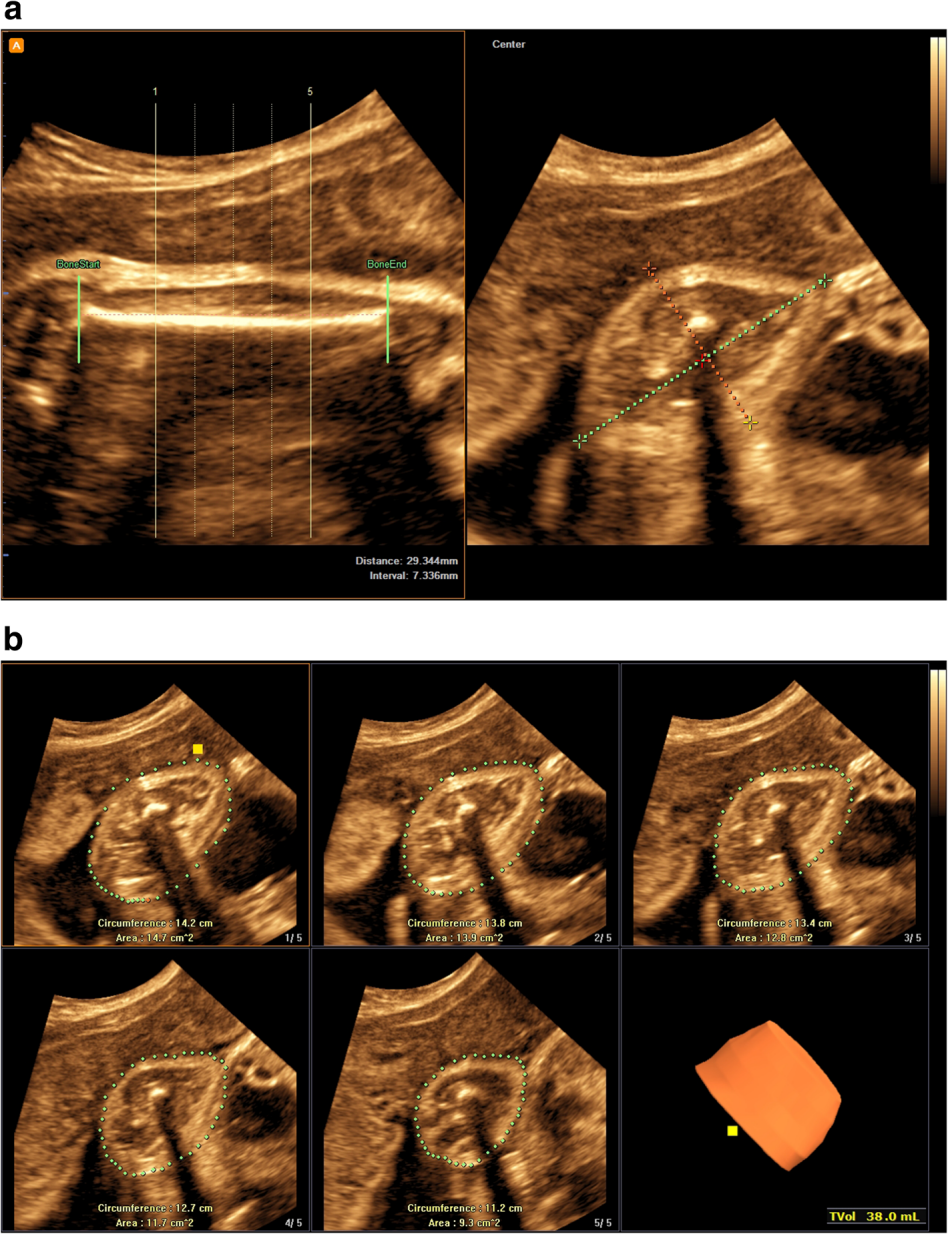
Fractional thigh volume measurement was calculated by novel software (5D Limb Vol; Samsung Medison). **a** step 1. The software identifies both ends of thigh limb automatically. Limb soft tissue borders are manually marked by examiner for short and long-axis diameters (red and green dotted lines) to initiate a computer-assisted edge detection algorithm. **b** step 2. The resulting fractional limb volume is divided into five subsections of equal length to allow automated tracing of surface contours from an axial view. The thigh volume and estimated fetal weight based BPD, AC and TVol were calculated automatically. BPD, biparietal diameter; AC, abdominal circumference; TVol, fractional thigh volume

Analysis was performed using the 5D Limb Vol software of the WS80A ultrasound system (5D Limb Vol; Samsung Medison). In the first step, the multiplanar mode simultaneously displayed the sagittal section of the long bone and the cross section of the corresponding limb center. The software automatically marked the positions of the long bone ends, and the examiner manually labeled the major and minor axis diameters on the generated cross section. In the second step, according to the determined long bone ends of the limbs, the system divided the middle 50% volume of the upper arm or the thigh of the fetus equally into five cross-sections and initiated the computer-assisted edge detection algorithm to automatically envelop the edges of the five limb cross-sections. Minor adjustments were possible after visual inspection of automated results. Finally, the fractional limb volume was automatically obtained (Fig. 1).

The fetal fractional limbs volume measurements were repeated by another examiner (ZN), who had only 1 year of obstetric ultrasound experience. The examiner was blinded to all previous measurements calculated.

### Fetal weight estimation

EFW was calculated by traditional 2D model using Hadlock’s formula [8] which included measurements of BPD, AC and FL. Hadlock formula: log10 weight = 1.335 – 0.0034 AC x FL + 0.0316 BPD + 0.0457 AC + 0.1623 FL.

3D fractional limb volume model used the Lee formulas [11] to estimate the fetal weight, using fractional arm volume (AVol) and fractional thigh volume (TVol) as parameters respectively.

Lee formulas: ln weight = 0.5046 + 1.9665 (ln BPD) − 0.3040 (ln BPD)^2^ + 0.9675 ln AC + 0.3557 ln AVol; ln weight =  −   0.8297 + 4.0344 (ln BPD) − 0.7820 (ln BPD)^2^ + 0.7853 (ln AC) + 0.0528 (ln TVol)^2^. Ln function in the formulas stands for the natural logarithm.

### Statistical analysis

We used the traditional 2D model and 3D fractional limb volume model to estimate fetal weight and calculated the systematic error and random error to evaluate the predictive performance of each model.

Accuracy, namely, the systematic error, is the mean percentage error (MPE) of the actual birth weight and estimated fetal weight, percentage error was defined as (estimated fetal weight - actual birth weight) divided by actual birth weight and multiplied by 100. Precision, namely, the random error, is expressed by the standard deviation (SD) of the percentage error. Count data are expressed as the mean ± SD. Differences between the models were compared using the Paired t test. The frequency data were analyzed using the Chi-square test. A two-way random effects model with absolute agreement was used to calculate the intraclass correlation coefficient (ICC) to indicate the measurement consistency between different examiners. The average score was been chosen as the ICC value. An ICC > 0.7 is commonly used to indicate sufficient reliability. To visually assess the systematic bias of different weight estimation models, we graphed scatter plots to describe the relationship of the difference between the estimated fetal weight and actual birth weight with the actual birth weight. Statistical analysis was performed using SPSS version 22.0 (IBM, Armonk, NY, USA). A two-sided *p*-value < 0.05 was considered statistically significant.

## Results

### Patient characteristics

A total of 56 pregnant women with a single fetus were examined by ultrasound within 7 days before delivery (mean 1.1 ± 1.9 days). Demographic and clinical data are shown in Table 1. Pregnancy complications included 6 cases of gestational hypertension, 10 cases of gestational diabetes, and 4 cases of immune system diseases (systemic lupus erythematosus and Sjögren’s syndrome).

**Table 1:** Demographic and clinical details of the study participants (*n* = 56)

Characteristics	Value
Maternal age (years)	31.7 ± 3.5
Maternal BMI (Kg/m^2^)	27.3 ± 3.5
GA at diagnosis (weeks)	39.6 ± 1.4
Ultrasound to delivery interval (days)	1.1 ± 1.9
Birth weight (g)	3393 ± 530
Birthweight category (g)	
< 3500 g	31
≥ 3500 g	25
Gravida	
1	23
2	20
3	12
4	1
Mode of delivery	
Vaginal delivery	38
Cesarean section	18
Sex of the neonate	
Male	28
Female	28

*GA* gestational age, *BMI* body mass index

### Comparison of 2D and 3D models

The average fetal weight estimated by the 2D Hadlock model was 3478 ± 467 g, and the MPE with the actual birth weight was 3.2 ± 8.9. The average fetal weight estimated by the 3D model using the AVol and TVol indicators were 3268 ± 467 g and 3250 ± 485 g, respectively, and the MPEs were − 3.3 ± 6.6 and − 3.9 ± 6.1, respectively. The 2D model tends to overestimate, and the 3D models tend to underestimate. The two models have similar absolute differences between the estimated fetal weight and actual birth weight (both < 4%), while the 3D models demonstrate better precision (lower random error, 6.6 vs. 8.9 and 6.1 vs. 8.9).

**Fig. 2:**
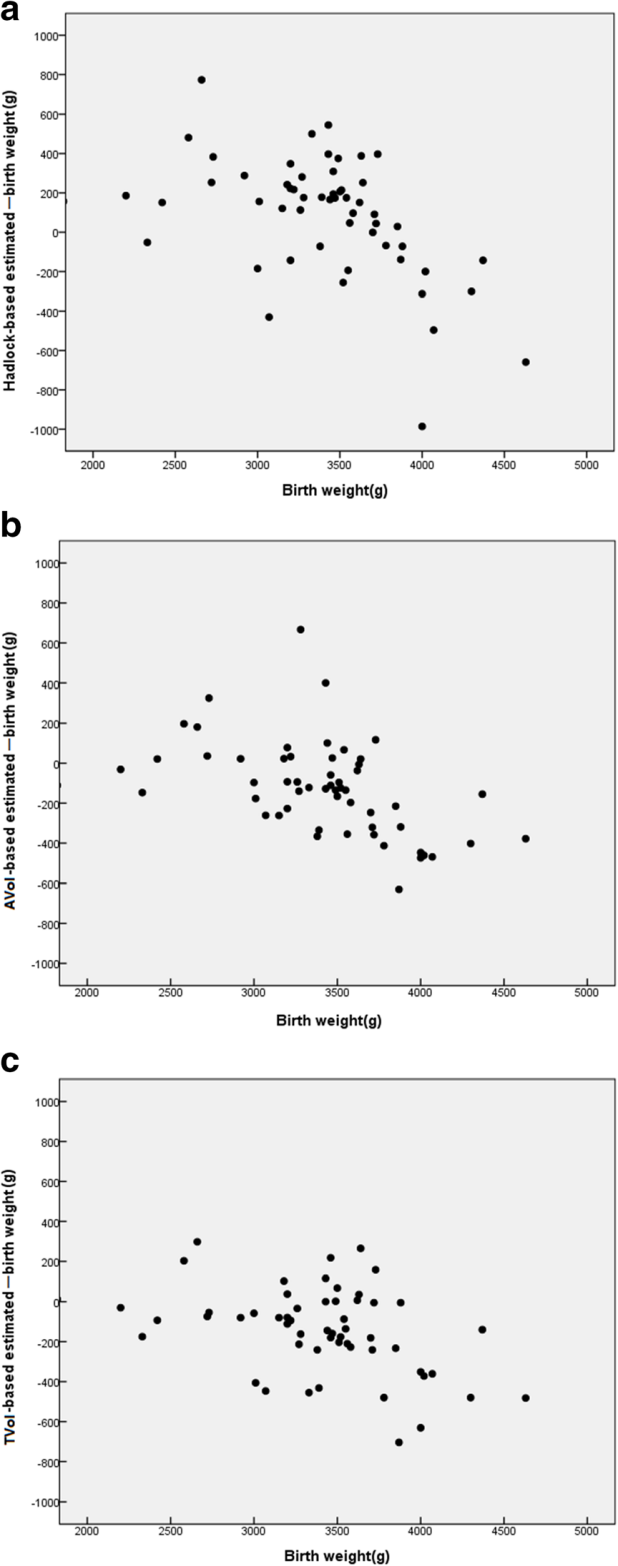
Scatter plots of differences between three models estimated fetal weight and actual birth weight, in relation to actual birth weight. **a**: Hadlock model; **b**: AVol model; **c**: TVol model. AVol, fractional arm volume; TVol, fractional thigh volume

Figure 2 shows the scatter plots of the actual birth weight and the difference between the estimated fetal weight and the actual body weight for the three models: Hadlock, AVol, and TVol. The plots clearly reveal that the differences from the actual birth weight for the AVol and TVol models show a narrower distribution around zero than Hadlock model. The difference between estimated fetal weight and actual birth weight shows a negative slope for all three models, suggesting that these models tend to underestimate the birth weight of large fetuses and overestimate the birth weight of small fetuses.

The numbers of estimated error ≤ 5% of actual birth weight in the Hadlock model, AVol mode, and TVol model were 19, 28, and 31, respectively, and the proportions were 33.9, 50.0, and 55.4%, respectively. In the comparison of the proportion of estimated error ≤ 5%, the TVol model was superior to the Hadlock model (*p* < 0.05), and there was no statistical difference between the Hadlock and AVol models (*p* = 0.085). The proportions of estimated error ≤ 10% in the Hadlock model, AVol model, and TVol model were 75.0, 82.1, and 82.1%, respectively, and there was no significant difference between the Hadlock model and the AVol model or TVol model (both *p* = 0.357) (Table 2).

**Table 2:** Comparison of two-dimensional and three-dimensional sonographic findings (*n* = 56)

Sonographic Findings	Hadlock	AVol	TVol
EFW (g)	3478 ± 467	3268 ± 467	3250 ± 485
MPE (%)	3.2 ± 8.9	- 3.3 ± 6.6	- 3.9 ± 6.1
EFW within 5% of BW, n (%)	19 (33.9)	28 (50.0)	31 (55.4)
EFW within 10% of BW, n (%)	42 (75.0)	46 (82.1)	46 (82.1)

*EFW* estimated fetal weight, *BW* birth weight, *MPE* mean percentage error, *AVol* fractional arm volume, *TVol* fractional thigh volume

Hadlock formula: log10 weight = 1.335–0.0034 AC x FL + 0.0316 BPD + 0.0457 AC + 0.1623 FL. Lee formulas: ln weight = 0.5046 + 1.9665 (ln BPD) − 0.3040 (ln BPD)^2^ + 0.9675 ln AC + 0.3557 ln AVol; ln weight = − 0.8297 + 4.0344 (ln BPD) − 0.7820 (ln BPD)^2^ + 0.7853 (ln AC) + 0.0528 (ln TVol)^2^

### Subgroup analysis

The prediction power of the three models for different and extreme birth weight neonates was compared by subgroup analysis (Table 3).

**Table 3:** Comparison of three antenatal ultrasonography models to predict different birth weight infants

Birth weight category	Hadlock	AVol	TVol
BW < 3500 g, *n* = 31			
MPE (%)	7.0 ± 7.8	- 0.8 ± 6.9	- 2.8 ± 5.9
EFW within 5% of BW, n (%)	6 (19.4)	18 (58.1)	20 (64.5)
EFW within 10% of BW, n (%)	22 (71.0)	27 (87.1)	26 (83.9)
BW ≥ 3500 g, *n* = 25			
MPE (%)	- 1.5 ± 8.1	- 6.4 ± 4.9	- 5.2 ± 6.0
EFW within 5% of BW, n (%)	13 (52.0)	10 (40.0)	11 (44.0)
EFW within 10% of BW, n (%)	20 (80.0)	19 (76.0)	20 (80.0)
BW ≤ 2500 g, *n* = 4			
MPE (%)	5.3 ± 5.1	- 3.2 ± 3.5	- 3.0 ± 3.5
BW ≥ 4000 g, *n* = 7			
MPE (%)	- 10.6 ± 7.3	- 9.6 ± 3.0	- 9.6 ± 3.7

*EFW* estimated fetal weight, *BW* birth weight, *MPE* mean percentage error, *AVol* fractional arm volume, *TVol* fractional thigh volume

Hadlock formula: log10 weight = 1.335–0.0034 AC x FL + 0.0316 BPD + 0.0457 AC + 0.1623 FL. Lee formulas: ln weight = 0.5046 + 1.9665 (ln BPD) − 0.3040 (ln BPD)^2^ + 0.9675 ln AC + 0.3557 ln AVol; ln weight = − 0.8297 + 4.0344 (ln BPD) − 0.7820 (ln BPD)^2^ + 0.7853 (ln AC) + 0.0528 (ln TVol)^2^

The MPEs of the AVol and TVol model for newborns (31 cases) with BW < 3500 g were − 0.8 ± 6.9 and − 2.8 ± 5.9, respectively, with significantly higher accuracy than that of the Hadlock model (7.0 ± 7.8) (both *p* < 0.05). The MPE of the Hadlock model for newborns (25 cases) with BW ≥ 3500 g was − 1.5 ± 8.1, indicating better accuracy than the AVol model and TVol model (− 6.4 ± 4.9, − 5.2 ± 6.0) (both *p* < 0.05). For newborns with BW < 3500 g, the proportions with estimated error ≤ 5% were 19.4, 58.1, and 64.5% in the Hadlock model, the AVol model, and the TVol model, respectively. Both AVol and TVol models were significantly superior to the Hadlock model (both *p* < 0.05). There was no significant difference in the proportion of estimated error ≤ 10% between the Hadlock model with the AVol and TVol models (*p* = 0.119, *p* = 0.224, respectively). For BW ≥ 3500 g, no statistical difference in the proportion of estimated errors ≤5% can be seen between the 2D model and the 3D model (Hadlock vs. AVol, *p* = 0.395; Hadlock vs. TVol, *p* = 0.571), and the difference in the proportion of estimated error ≤ 10% between two types of models was not significant either (Hadlock vs. AVol, *p* = 0.733; Hadlock vs. TVol, *p* = 1.000).

Four low-birth-weight newborns (BW ≤ 2500 g) were clinically diagnosed as late-onset FGR, with an average birth weight of 2193 g. The MPEs of the AVol and TVol models for estimating the FGR fetuses were − 3.2 ± 3.5 and − 3.0 ± 3.5, respectively, with higher accuracy than the Hadlock model (5.3 ± 5.1) (both *p* < 0.05). Seven macrosomic infants (BW ≥ 4000 g) had an average birth weight of 4199 g. With regard to the estimated weight of macrosomia, there was no statistical difference between the Hadlock model with the AVol and TVol models (*p* = 0.741, *p* = 0.763, respectively).

### Inter-observer reliability

The inter-observer reliability of measuring the fetal limb volumes using the 5D Limb Vol software tool were high. The ICC of AVol was 0.921 (95% CI, 0.868–0.953), and the ICC of TVol was 0.963 (95% CI, 0.937–0.978).

## Discussion

Our study applied the 5D limb Vol software to the Chinese population alone. The results showed that there was no statistical difference in the accuracy of fetal weight estimation between the 3D fractional limb volume models and the traditional Hadlock 2D model, while the 3D models had low random error and higher precision. In the population of this study, using the AVol model and the TVol model, the differences between the estimated weight and actual birth weight of ≤5% were achieved for half of the fetuses, with proportions of 50.0 and 55.4%, respectively, which were similar to the previous study results [11, 13] and significantly superior to the 2D Hadlock model (33.9%).

Precise fetal weight estimation means that repeatable results are provided, which is extremely important for daily obstetric care. A review of the medical literature indicates that fractional limb volume has been used for EFW in many countries. Although the models used and sample size are different, many studies also have shown that the introduction of fractional limb volume improve the weight estimation precision of normal fetuses in the third trimester, which are similar to our research conclusions (Table 4). Therefore, 3D fractional limb volume model to estimate fetal weight may better meet the clinical requirements for the accuracy of fetal weight prediction.

**Table 4:** Summary of fetal weight estimation studies based on fractional limb volume

First author	Sample size	Gestational age (weeks)	Birth weight (g)	Main comments	MPE (%)	EFW within 5% of BW (%)	EFW within 10% of BW (%)
Lee (2001) [10]	100 for model derivation,30 for validation	39.2 ± 1.2	3643 ± 574	The model containing AC and TVol had the best predictive capacity for fetal weight.	2.3 ± 6.6	66.67	96.67
Lee (2009) [11]	271	scanned within 4 days of delivery	235–5790	Two models, which used the natural logarithms of BPD, AC, AVol and TVol provided the most precise weight estimations.	0.18 ± 6.6(AVol) 0.12 ± 6.6(TVol)	50.4(AVol) 57.3(TVol)	89.8(AVol) 84.1(TVol)
Yang (2011) [12]	100 for model derivation,190 for validation	38.7 ± 3.1	3202 ± 360	The prediction model using TVol, FL, AC and BPD provided the most precise birth-weight estimation.	0.23 ± 4.68	69.5	95.3
Lee (2013) [13]	164	37.1 ± 4.1	3057 ± 1102	Optimal model performance resulted from using a combination of BPD, AC and TVol.	1.9 ± 6.6	55.1	86.5
Mack(2017) [14]	50	39.1 ± 1.4	3335	Automated fractional limb volume measurements improved the precision of weight predictions in third-trimester fetuses.	−9.1 ± 5.1(AVol) − 5.2 ± 5.2(TVol)	Not provided	
Sharma(2019) [15]	100 for model derivation,31 for validation	38.0 ± 0.9	3039.2 ± 42	The best fit model for fetal weight estimation comprised BPD, HC, AC, and TVol.	0.624 ± 8.075	70.2	91.6
Wu (current)	56	39.6 ± 1.4	3393 ± 530	Automated 3D fractional limb volume model improved the accuracy of weight estimation in most third-trimester fetuses.	−3.3 ± 6.6(AVol) − 3.9 ± 6.1(TVol)	50.0(AVol) 55.4(AVol)	82.1(both of AVol and TVol)

*EFW* estimated fetal weight, *BW* birth weight, *MPE* mean percentage error, *AVol* fractional arm volume, *TVol* fractional thigh volume

The subgroup analysis of birth weight showed that the AVol and TVol models had high accuracy for the weight estimation of newborns < 3500 g in weight, and the proportions with an estimated weight difference of ≤5% were 58.1 and 64.5%, respectively, significantly higher than that of the Hadlock model. In clinical practice, neonatal birth weight often does not exceed 3500 g. Therefore, it can be speculated that acquisition of high-quality limb volumes with 3D sonography for fetal weight estimation can be feasible in these nonmacrosomic fetuses.

There were four newborns with BW ≤ 2500 g in our group. Applying AVol and TVol models to estimate fetal weight before delivery had high accuracy, and the differences with birth weight were less than 5%. All of the low-birth-weight newborns in this study were diagnosed late-onset FGR and were timely delivered at 34 to 38 weeks after indicating low weight by the 3D models. Accurate estimation of fetal weight by prenatal ultrasound is an important aspect of the identification and diagnosis of FGR; however, the detection rate of late-onset FGR is only 23 to 51% [17, 18]. Simcox et al. also showed that using the TVol to estimate fetal weight increased the detection rate of 34- to 36-week late-onset FGR [19]. Therefore, our results indicate that the application of 3D fractional limb volume model to estimate fetal weight may help detect fetal growth and development abnormalities in time and improve the detection of low-weight fetuses or late-onset FGR.

Different opinions have been offered on the weight estimation of macrosomia [20–23]. Gibson et al. have shown that TVol is the optimum ultrasound index for predicting the weight of macrosomia [23]. A recent multicenter study also showed that there was a clear trend for automated fractional limb models to provide improved weight estimates in larger fetuses with BW of greater than 4000 g [24], while other studies have shown that the accuracy of different weight estimation models decreases with increasing fetal weight [25, 26]. The data in our study showed that there was no significant difference in the accuracy of weight estimation between the 3D model and the 2D model for seven cases of macrosomia (BW ≥ 4000 g). This study also showed that the estimation accuracy of the 3D model for newborns with BW ≥ 3500 g was not as good as that for newborns with BW < 3500 g. The possible reason is that the soft tissue of the fetuses more than 3500 g of estimated weight could be compressed in the uterus, which affects the recognition of the limb boundary, thus limits the acquisition and analysis of the 3D volume data to some extent. Therefore, the accurate estimation of large fetuses using the 3D fractional limb volume requires the accumulation of further experience, and better indicators to quantify fetal soft tissue and developing new models to predict neonatal adiposity.

In most previous studies, the primary limitation of using 3D ultrasound to measure fetal limb volume was to manually trace the limb boundaries of the five cross-sections [10–13, 15], which is time consuming and leads to limited clinical application. The 5D limb vol software in this study uses computer-aided measurement technology, which can automatically trace the boundary of the limb in cross-sections to obtain fetal limb volume and estimated fetal weight, and manual adjustment can be conducted if needed. The measurement time of a limb volume using this software is only about 30–40 s, while it took about 2 min to obtain the limb volume by manually tracing the limb boundary. Moreover, the optimaluse of sonographic fetal biometry depends on an operator-dependent procedure that requires careful attention to the measurement protocol and the use of appropriate imaging equipment. Operators with inexperience are likely to cause large measurement errors. Our study analyzed the use of 5D limb vol software by examiners with different experience in obstetric ultrasound to evaluate the consistency of fetal AVol and TVol. The results showed that relatively good agreement of the AVol and TVol measurements between the novice and experienced operators, indicating that the use of automatic limb volume software to estimate fetal weight is conducive for reducing operator dependence. Automated fractional limb volume measurements is easy to operate and timesaving, and has broad clinical application prospects.

Small sample size is the main limitation of our research, especially the numbers of low-birth-weight newborns and macrosomia. The accuracy of estimating fetuses with extreme birth weights using fractional limb volume awaits further study. Additionally, there were no cases with extremely low volumes of amniotic fluid in the target population, and it is thereby impossible to determine the effect of amniotic fluid volume on the image quality of 3D limb volume.

## Conclusions

In summary, this pilot study used automated limb volume estimation software to prospectively evaluate the weight of Chinese fetuses in the third trimester. Prediction accuracy of the 3D model for neonatal BW, particularly < 3500 g was higher than that of the traditional 2D model, suggesting potentially good clinical application prospects of the 3D model.

## Data Availability

All data generated or analysed during this study are included in this published article (Results section and tables within the manuscript).

## Abbreviations

2D: Two-dimensional
3D: Three-dimensional
AVol: Fractional arm volume
TVol: Fractional thigh volume
BPD: Biparietal diameter
AC: Abdominal circumference
FL: Femur length
MPE: Mean percentage error
EFW: Estimated fetal weight
BW: Birth weight
FGR: Fetal growth retardation
ICC: Intraclass correlation coefficient
g: Grams
